# Right ventricular function in pulmonary hypertension and obesity: a cross-sectional cohort study with survival follow-up

**DOI:** 10.1007/s00392-025-02682-9

**Published:** 2025-06-24

**Authors:** B. Egenlauf, M. Braun, V. Schiffer, A. M. Marra, P. Xanthouli, S. Harutyunova, C. A. Eichstaedt, C. Erbel, R. Schell, F. Linden, E. Grünig, Nicola Benjamin

**Affiliations:** 1 Centre for Pulmonary Hypertension, Thoraxklinik Heidelberg gGmbH at Heidelberg University Hospital, Röntgenstraße 1, 69126 Heidelberg, Germany; 2 Translational Lung Research Centre Heidelberg (TLRC), Member of the German Centre for Lung Research (DZL), Heidelberg, Germany; 3 Department of Pneumology and Critical Care Medicine, Thoraxklinik Heidelberg gGmbH at Heidelberg University Hospital, Heidelberg, Germany; 4 Department of Internal Medicine V: Haematology, Oncology and Rheumatology, University Hospital Heidelberg, Heidelberg, Germany; 5 Laboratory for Molecular Genetic Diagnostics, Institute of Human Genetics, Heidelberg University, Heidelberg, Germany; 6 Department of Translational Medical Sciences, “Federico II” University and School of Medicine, Naples, Italy; 7 Department of Cardiology, Pneumology and Angiology at University Hospital, Heidelberg University Hospital, Heidelberg, Germany

**Keywords:** Pulmonary hypertension, Obesity paradox, Right ventricular function, Cardiac output, Echocardiography, Right-heart catheterization, Survival

## Abstract

**Background:**

Obesity or underweight can complicate and aggravate symptoms and progression of right heart failure in patients with pulmonary arterial hypertension (PAH). This study investigates the influence of different body mass index (BMI) categories on right heart function and outcome in PAH patients.

**Methods:**

In this cross-sectional study with survival follow-up (mean follow-up 3.1 ± 2.6 years, median 2.7 years), clinical measures such as WHO-functional class and invasively measured hemodynamic parameters at initial diagnosis of PAH were compared between different BMI groups.

**Results:**

Out of 2055 data sets, 755 patients with PAH (62.5% female) were eligible for the study (65 ± 15 years, 44.9% idiopathic PAH, 64.8% WHO functional class III or IV). Out of them 15 patients (1.99%) were underweight (BMI < 18 kg/m^2^), 248 (32.85%) patients had a normal weight (BMI 18.5–25 kg/m^2^), 256 (33.91%) were overweight (BMI > 25 to 30 kg/m^2^) and 236 patients (31.26%) were classified as obese (BMI > 30 kg/m^2^). Worst survival was denoted for patients with BMI < 18.5 kg/m^2^, best survival for BMI > 25 to 30 kg/m^2^. Cardiac output (CO) significantly differed between BMI groups (p < 0.0001, R = 0.268) and sex. In multivariable age-adjusted survival analysis, BMI-status, sex and right ventricular function were identified as independent predictors of survival.

**Conclusions:**

This is the first study to assess RV function with regard to BMI status and survival in PAH. The study underlines the importance of the parameter body weight in the clinical management of PAH patients. It provides important insights in the relations of BMI and CO and documented significant gender differences.

**Graphical abstract:**

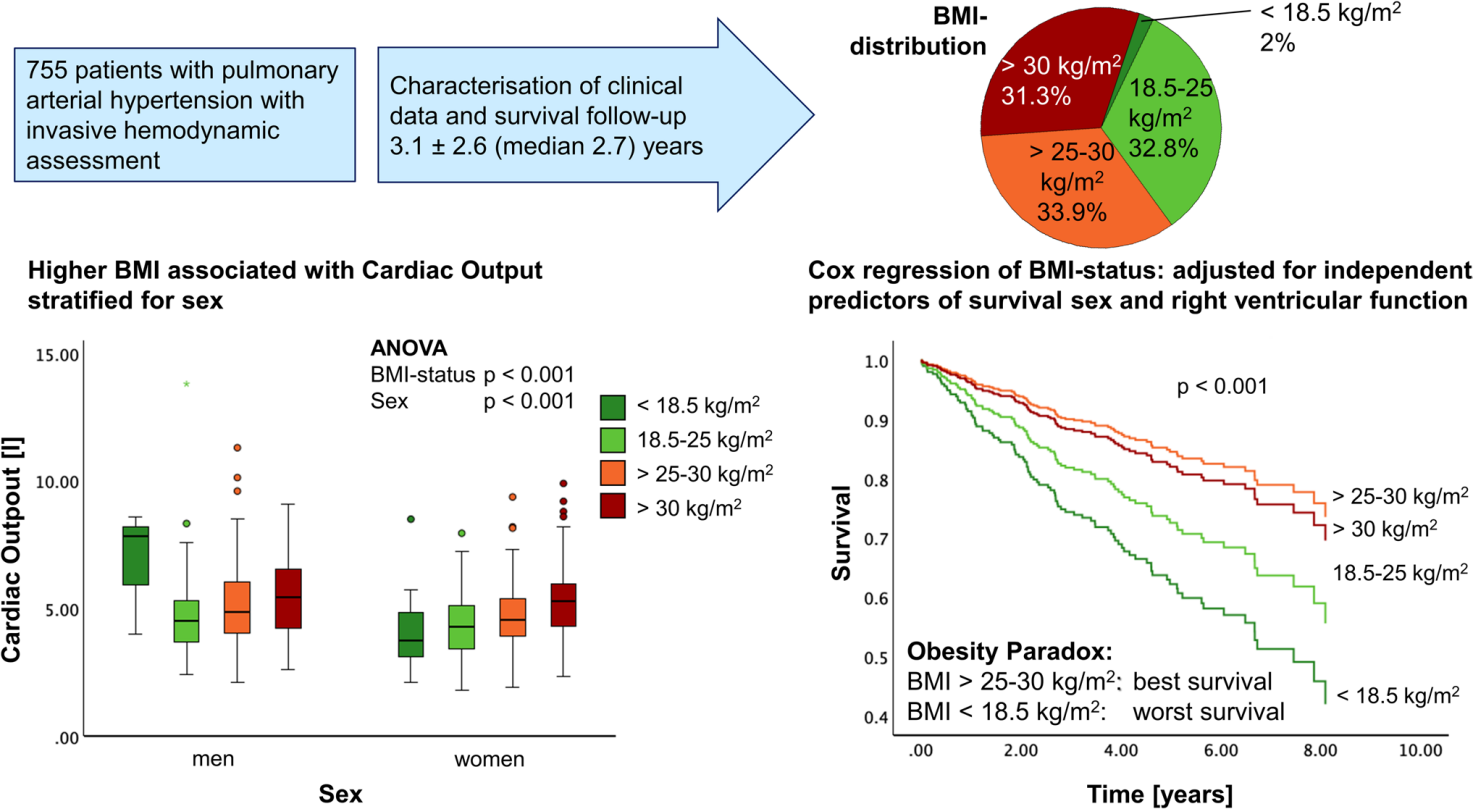

**Supplementary Information:**

The online version contains supplementary material available at 10.1007/s00392-025-02682-9.

## Introduction

Pulmonary arterial hypertension (PAH) is a rare disease characterised by progressive pulmonary vascular remodelling, resulting in right ventricular failure which can lead to death [1]. The phenotype of the disease has been changed within the last decades. Increasingly, patients diagnosed with PAH are older with more comorbidities as overweight or obesity [2]. Obesity can influence the morphology and function of the heart and can result in heart insufficiency or left heart hypertrophy [3], as well as increased right heart size, end diastolic volume and stroke volume (SV), while the ejection fraction is decreased [4]. Obesity is a known cardiovascular risk factor and comorbidity of PH [5]. Analysis of the REVEAL-registry showed that 33% of PH-patients are obese [6]. On the other hand, obesity seems to be a protective factor in several chronic cardiovascular conditions, such as left-sided heart failure, a phenomenon called “obesity paradox” [7]. Some authors have recognised the possibility of it being a statistical artefact or confounding factors being responsible. Some clinical studies in PH support the hypothesis that increased BMI might have a protective effect [8, 9]. The study of Weatherald et al. 2021 analysing the French PH-registry reported significantly worse haemodynamic parameters in their obese group compared to their nonobese group [10]. So far, there is limited clinical knowledge on the association between obesity and right heart function in patients with PAH [9]. There are almost no studies on underweight and hemodynamic consequences in PH. Therefore, the current work sought to determine the association of body mass index with right heart hemodynamic parameters and survival in PAH-patients.

## Methods

### Study population

Adults who were initially diagnosed with PAH from May 2017 to July 2022 at the Centre for pulmonary hypertension of the Thoraxklinik at Heidelberg University Hospital (Germany) were retrospectively included in this study. The patients underwent a routine diagnostic work up, including extensive laboratory, RHC, echocardiography, lung function, blood gas analysis, spirometry and 6-min-walking-distance (6MWD). All data was collected at time of diagnosis by RHC. Excluded were underage patients, patients suffering from acute cardiac decompensation and patients with congenital heart anomaly.

PAH patients (defined by mean pulmonary arterial pressure (mPAP) > 20 mmHg, pulmonary arterial wedge pressure (PAWP) ≤ 15 mmHg and pulmonary vascular resistance (PVR) ≥ 3 WU) were then selected for further investigation, while patients with other forms of PH were excluded from comparative analysis.

The ethics committees of the Medical Faculty Heidelberg (internal reference S-407/2022) had no objection against the conduct of the trial. The study complies with the Declaration of Helsinki in its current version.

### Study design

This was a retrospective cross-sectional study with survival follow-up (3.1 ± 2.6 years, median 2.7 years). Survival data of patients was collected according to routine follow-up records. To evaluate the impact of obesity on CO, the study population was categorised into four groups according to their BMI. These groups were defined according to World Health Organization (WHO) standard as follows: underweight (BMI < 18.5 kg/m^2^), normal (BMI 18.5–25 kg/m^2^), overweight (BMI 25–30 kg/m^2^) and obese (BMI > 30 kg/m^2^) [11]. CO, hyperdynamic status and clinical characteristics were then described and compared between BMI-groups. Impact of BMI-status on survival was investigated. All data was checked for plausibility, consistency and completeness and then pseudonymised.

### Trial assessments

Clinical data were obtained from routine diagnostics at initial diagnosis of PAH. Assessments included medical history, concomitant medications, invasive measurement of hemodynamics by RHC, echocardiography, 6MWD, body plethysmography, diffusion capacity for carbon monoxide (DLCO) and blood gas analysis, WHO functional class and laboratory parameters (especially N-terminal pro brain natriuretic peptide (NT-proBNP), glomerular filtration rate, urea, serum creatinine, Troponin-T, C-reactive protein (CRP), and leukocytes) according to the standards of the site.

*Echocardiography* Resting transthoracic echocardiography Doppler examinations were performed by experienced cardiac sonographers (BE, PX, SH) with commercially available equipment (Vivid E95, GE Healthcare, Milwaukee, Wisconsin) according to standardised protocol as described previously. Specific indices included right and left heart dimensions, diameter of vena cava inferior, systolic pulmonary atrial pressure (sPAP), left ventricular eccentricity index (LV-EI), tricuspid annular plane systolic excursion (TAPSE) and TAPSE/sPAP. Right ventricular function and signs for left ventricular dysfunction or valvular heart diseases were assessed by experienced sonographers.

*Right heart catheterization* Hemodynamic values were obtained from the centre’s database. RHC was performed in a standardised way in a supine position using a transjugular access with a triple-lumen 7F-Swan-Ganz thermodilution catheter. Patients had been examined on a variable load supine bicycle ergometer by experienced investigators (BE, PX, SH). The zero reference point for pressure recordings was set at ½ of the thoracic diameter below the anterior thorax surface [12]. Pressures were continuously recorded and averaged over several respiratory cycles during spontaneous breathing. CO was determined by thermodilution at least in triplicate with a variation of less than 10% between the measured values.

Parameters measured by RHC included central venosus/right atrial pressure, systolic/diastolic/mean pulmonary atrial pressure (s/d/mPAP), PAWP and CO. PVR, pulmonary arterial compliance (PAC), SV, stroke volume index (SVI) and cardiac index (CI) was calculated offline.

### Hyperdynamic status

To elucidate the so called “obesity paradox” (if it exists) we also wanted to analyse if obesity may lead to a hyperdynamic right ventricular pump function. For this analysis we used the following definitions for hyperdynamic status, which are currently used in clinical practice. Patients with a CO > 3.9 × body surface area (BSA) are defined to be hyperdynamic [13], leading to a role of thumb for hyperdynamic status as second definition with a CO > 8 l/min. This definition is often used in clinical practice as rule of thumb [13]. To consider the differences of body dimension, the calculation including body surface area was used for further analysis.

### Statistical methods

Data are described as mean ± standard deviation (SD) with 95% confidence interval of the mean or n and % for frequency data. The primary end point of this study was the comparison of CO between different BMI-groups. Difference of CO between BMI groups was compared by ANOVA and post-hoc tests comparing the different groups. To account for outliers, a nonparametric ANOVA (Kruskal–Wallis test) was performed. ANOVA analyses were stratified to identify the influence of sex (ANCOVA).

Correlation analysis of BMI and clinical parameters was performed by Pearson correlation with > 0.6 defining a strong correlation, 0.3–0.6 a moderate correlation and < 0.3 a weak correlation. Differences of clinical characteristics between different BMI groups were compared by two-tailed student’s *t* tests. For analysis of categorial variables the chi-square test was used.

For exploratory analysis of survival, baseline was defined as the date of initial diagnosis of PAH. Kaplan–Meier analysis and age-adjusted Cox regression analysis were used to determine the influence of BMI on survival. Multivariable age-adjusted Cox regression was performed to identify independent predictors of survival with right ventricular function at baseline, BMI status, sex, CO and CI. Due to the wish of one reviewer, an age- and systolic blood pressure adjusted Cox regression analysis of BMI status was performed.

No imputation of missing data was performed. All analyses were performed using IBM SPSS 27 (SPSS Statistics V.27, IBM Corporation, Somers, New York, USA).

### Ethics statement

The local Ethics Committee of the Medical Faculty of Heidelberg University (internal number S-407/2022) had no objection against the conduct of the trial. All data were pseudonymised. The study complied with the Declaration of Helsinki in its current version.

## Results

### Included patients and baseline characteristics (Table 1)

**Table 1:** Characteristics of the study cohort

Parameter [unit]	Whole cohort		
	(N = 755)		
	N^a^	Mean ± standard deviation	95% confidence interval
Female sex no. [%]		472 (62.5%)	
Age [years]		65 ± 15	(63–66)
Height [cm]		167.22 ± 9.23	(166.56–167.88)
Weight [kg]		78.70 ± 19.60	(77.30–80.10)
BMI [kg/m^2^]		28.07 ± 6.39	(27.72–28.53)
HR [bpm]	754	74.83 ± 15.04	(73.75–75.90)
Systolic blood pressure [mmHg]	750	136.43 ± 22.13	(134.84–138.01)
Diastolic blood pressure [mmHg]	750	73.35 ± 10.46	(72.60–74.10)
Diagnosis			
IPAH		540 (71.5%)	
HPAH		18 (2.4%)	
DPAH		2 (0.3%)	
APAH		195 (25.8%)	
Diabetes mellitus		160 (21.2%)	
Type 1		7 (0.9%)	
Type 2		152 (20.1%)	
Type 3		1 (0.1%)	
WHO FC no [%]			
I		6 (0.9%)	
II		238 (34.3%)	
III		386 (55.6%)	
IV		64 (9.2%)	
BMI-Status			
< 18.5		15 (2.0%)	
18.5–25		248 (32.9%)	
25–30		256 (33.9%)	
> 30		236 (31.3%)	
Hemodynamics at rest			
mPAP [mmHg]		35.38 ± 13.30	(34.43–36.33)
PAWP [mmHg]		10.56 ± 3.00	(10.35–10.78)
CO [l/min]	754	4.89 ± 1.47	(4.78–4.99)
CI [l/min/m^2^]	754	2.62 ± 0.73	(2.57–2.67)
PVR [WU]	754	5.79 ± 4.33	(5.48–6.10)
PAC [ml/mmHg]	753	2.41 ± 1.45	(2.31–2.51)
Echocardiography at rest			
sPAP [mmHg]	706	53.54 ± 20.84	(52.00–55.08)
RA area [cm^2^]	682	18.90 ± 7.24	(18.35–19.44)
RV area [cm^2^]	689	20.53 ± 6.55	(20.04–21.02)
TAPSE [mm]	691	2.14 ± 0.59	(2.10–2.18)
Lung function			
FVC [%]	718	80.50 ± 22.07	(78.89–82.12)
FEV1 [%]	730	77.36 ± 22.14	(75.75–78.97)
TLC [%]	725	95.17 ± 19.51	(93.74–96.59)
DLCO [%]	630	52.85 ± 24.42	(50.94–54.76)
6MWD			
6MWD [m]	453	356.15 ± 123.35	(344.76–367.54)
Laboratory			
NT-proBNP [pg/ml]	642	2113.84 ± 6568.37	(1604.79–2622.89)
GFR CDK-EPI [ml/min/1.73m^2^]	751	70.81 ± 24.88	(69.02–72.59)
Urea [mg/dl]		45.28 ± 28.38	(43.25–47.31)
Serum creatinine [mg/dl]		1.06 ± 0.53	(1.02–1.10)
TropT [µg/l]	591	19.33 ± 19.98	(17.71–20.94)
CRP [mg/dl]	432	13.97 ± 19.79	(12.10–15.84)
Leukocytes [/nl]	753	8.06 ± 3.66	(7.80–8.33)

*APAH* associated PAH, *BMI* Body Mass Index, *CI* cardiac index, *CO* cardiac output, *CRP* C-reactive protein, *DPAH* drug and toxin associated PAH, *DLCO* diffusing capacity of the lung for carbon monoxide, *FEV1* forced expiratory volume in first second, *FVC* forced vital capacity, *GFR CDK-EPI* estimated glomerular filtration rate by Chronic Kidney Disease Epidemiology Collaboration Formula, *HPAH* heritable pulmonary arterial hypertension, *HR* heart rate, *IPAH* idiopathic pulmonary arterial hypertension, *mPAP* mean pulmonary arterial pressure, *NT-proBNP* N-terminal pro brain natriuretic peptide, *PAH* pulmonary arterial hypertension, *PAC* pulmonary arterial compliance, *PAWP* pulmonary arterial wedge pressure, *PVR* pulmonary vascular resistance, *RA* right atrial, *RV* right ventricular, *sPAP* systolic pulmonary arterial pressure, *SD* standard deviation, *TAPSE* tricuspid annular plane systolic excursion, *TLC* total lung capacity, *TropT* Troponin-T, *WHO FC* World Health Organization functional class, *WU* Wood Units

^a^Sample size is given in case of missing values

Between May 2017 and July 2022, a total of 2055 data sets were identified. After checking inclusion and exclusion criteria and considering the PH groups, 755 patients with PAH were statistically evaluated (Table 1): 472 females (62.5%), 540 (71.5%) idiopathic PAH (IPAH), 55.6% WHO FC III, 9.2% WHO FC IV, mean age 65 ± 15 years, mean height 167.22 ± 9.23 cm, mean weight 78.70 ± 19.60 kg, mean BMI 28.07 ± 6.39 kg/m^2^.

Fifteen patients (1.99%) were underweight, while the other patients had a normal weight (n = 248, 32.9%), were overweight (n = 256, 33.9%), or were classified as obese (n = 236, 31.3%, Table 2).

**Table 2:** Characteristics of the BMI group

BMI	< 18.5		18.5–25		25–30		> 30	
	(N = 15)		(N = 248)		(N = 256)		(N = 236)	
Parameter [unit]	N^a^	Mean ± SD or n (%)	N^a^	Mean ± SD or n (%)	N^a^	Mean ± SD or n (%)	N^a^	Mean ± SD or n (%)
Female sex no. [%]		12 (80.0%)		153 (63.9%)		156 (61.7%)		145 (62.2%)
Age [years]	15	55 ± 21	248	65 ± 16	256	65 ± 14	236	64 ± 13
Height [cm]	15	164.80 ± 6.11	248	167.07 ± 8.66	256	167.53 ± 9.85	236	167.20 ± 9.52
Weight [kg]	15	45.90 ± 6.30	248	62.60 ± 8.20	256	77.00 ± 10.00	236	99.50 ± 16.50
BMI [kg/m^2^]	15	16.88 ± 1.93	248	22.37 ± 1.69	256	27.34 ± 1.44	236	35.58 ± 5.11
BSA [m^2^]	15	1.48 ± 0.12	248	1.70 ± 0.15	256	1.86 ± 0.18	236	2.07 ± 0.20
HR [bpm]	15	86.20 ± 20.26	248	75.08 ± 15.57	255	75.09 ± 15.78	236	73.56 ± 12.89
Systolic blood pressure [mmHg]	14	127.29 ± 18.93	248	131.90 ± 21.96	255	138.69 ± 22.62	233	139.32 ± 21.14
Diastolic blood pressure [mmHg]	14	70.21 ± 11.89	248	70.43 ± 9.09	255	75.29 ± 11.22	233	74.53 ± 10.21
Diagnosis								
IPAH		8 (53.3%)		171 (69.0%)		180 (70.4%)		181 (76.7%)
HPAH		0		8 (3.2%)		7 (2.7%)		3 (1.3%)
DPAH		0		0		0		2 (80%)
APAH		7 (46.7%)		69 (27.8%)		69 (27.0%)		50 (21.0%)
Diabetes mellitus				18 (7.3%)		48 (18.8%)		91 (38.6%)
Typ 1		3 (20.0%)		2 (0.8%)		1 (0.4%)		4 (1.7%)
Typ 2		2 (13.3%)		16 (6.5%)		47 (18.4%)		87 (36.9%)
Typ 3		1 (6.70%)		0		0		0
WHO FC no [%]								
I		0		5 (2.2%)		0		1 (0.5%)
II		4 (30.8%)		84 (36.5%)		93 (39.9%)		57 (26.1%)
III		5 (38.5%)		117 (50.9%)		124 (53.2%)		140 (64.2%)
IV		4 30.8%)		24 (10.4%)		16 (6.9%)		20 (9.2%)
Hemodynamics at rest								
mPAP [mmHg]	15	40.00 ± 15.38	248	34.70 ± 12.72	256	35.69 ± 13.51	236	35.46 ± 13.56
PAWP [mmHg]	15	9.93 ± 3.10	248	9.96 ± 3.28	256	10.54 ± 2.98	236	11.26 ± 2.54
CO [l/min]	15	4.72 ± 2.07	248	4.46 ± 1.38	256	4.91 ± 1.48	235	5.33 ± 1.39
CI [l/min/m^2^]	15	3.17 ± 1.30	248	2.62 ± 0.75	256	2.63 ± 0.72	235	2.57 ± 0.64
PVR [WU]	15	7.19 ± 3.83	248	6.36 ± 4.74	256	5.78 ± 4.33	235	5.10 ± 3.78
PAC [ml/mmHg]	15	1.88 ± 1.10	248	2.13 ± 1.21	256	2.33 ± 1.43	236	2.80 ± 1.63
Echocardiography at rest								
sPAP [mmHg]	14	61.43 ± 22.82	238	54.55 ± 20.86	237	54.11 ± 21.53	217	51.31 ± 19.78
RA area [cm^2^]	14	16.43 ± 7.50	229	18.43 ± 7.93	230	19.22 ± 7.10	209	19.21 ± 6.55
RV area [cm^2^]	14	17.07 ± 5.12	230	19.49 ± 6.90	233	20.79 ± 6.60	212	21.60 ± 5.95
TAPSE [mm]	15	19.00 ± 3.78	228	20.42 ± 5.89	231	21.98 ± 5.94	217	21.98 ± 5.84
TAPSE/sPAP [mm/mmHg]	14	3.62 ± 1.81	225	4.42 ± 2.40	228	5.39 ± 7.09	213	5.05 ± 2.48
Lung function								
FVC [%]	12	62.81 ± 14.21	234	82.73 ± 21.68	250	80.66 ± 22.00	222	78.93 ± 22.45
FEV1 [%]	13	58.18 ± 20.21	237	78.08 ± 20.91	252	78.30 ± 22.76	228	76.65 ± 22.40
TLC [%]	13	95.63 ± 23.62	237	97.39 ± 19.63	251	93.91 ± 20.57	224	94.20 ± 17.77
DLCO [%]	9	41.22 ± 14.28	211	50.01 ± 26.25	215	52.17 ± 23.12	195	57.20 ± 23.55
6MWD								
6MWD [m]	8	375.75 ± 97.13	157	372.75 ± 137.87	162	366.44 ± 109.86	126	321 ± 116.04
Laboratory								
NT-proBNP [pg/ml]	8	3439.00 ± 2819.18	210	3034.93 ± 9247.61	228	2025.56 ± 6102.44	196	1175.56 ± 2155.58
GFR CDK-EPI [ml/min/1.73m^2^]	15	81.35 ± 36.26	245	73.37 ± 25.22	256	70.90 ± 23.52	235	67.36 ± 24.76
urea [mg/dl]	15	47.13 ± 44.49	248	42.70 ± 26.48	256	44.79 ± 26.74	236	48.41 ± 30.59
Serum creatinine [mg/dl]	15	1.16 ± 1.14	248	1.01 ± 0.49	256	45,778 ± 0.51	236	1.12 ± 0.52
TropT [µg/l]	8	21.99 ± 15.58	195	20.62 ± 24.31	210	18.83 ± 20.18	178	18.37 ± 13.74
CRP [mg/dl]	12	24.94 ± 36.60	121	12.76 ± 18.54	125	15.91 ± 25.17	174	12.65 ± 13.55
Leukocytes [/nl]	15	8.97 ± 3.98	248	8.15 ± 5.20	255	7.86 ± 2.69	235	8.14 ± 2.37

*APAH* associated PAH, *BMI* Body Mass Index, *BSA* Body Surface Area, *CI* cardiac index, *CO* cardiac output, *CRP* C-reactive protein, *DPAH* drug and toxin associated PAH, *DLCO* diffusing capacity of the lung for carbon monoxide, *FEV1* forced expiratory volume in first second, *FVC* forced vital capacity, *GFR CDK-EP*I estimated glomerular filtration rate by Chronic Kidney Disease Epidemiology Collaboration Formula, *HPAH* heritable pulmonary arterial hypertension, *HR* heart rate, *IPAH* idiopathic pulmonary arterial hypertension, *mPAP* mean pulmonary arterial pressure, *NT-proBNP* N-terminal pro brain natriuretic peptide, *PAH* pulmonary arterial hypertension, *PAC* pulmonary arterial compliance, PAWP pulmonary arterial wedge pressure, *PVR* pulmonary vascular resistance, *RA* right atrial, *RV* right ventricular, *sPAP* systolic pulmonary arterial pressure, *SD* standard deviation, *TAPSE* tricuspid annular plane systolic excursion, *TLC* total lung capacity, *TropT* Troponin-T, *WHO FC* World Health Organization functional class, *WU* Wood Units

^a^Sample size is given in case of missing values

Patients presented with severely impaired right heart function with mean CO of 4.89 ± 1.47 (median 4.75) l/min, mean CI of 2.62 ± 0.73 (median 2.56) l/min/m^2^, mean PVR of 5.79 ± 4.33 (median 4.21) WU, mean PAC of 2.41 ± 1.45 (median 2.14) ml/mmHg and mean TAPSE of 2.14 ± 0.59 (median 2.10) cm (Table 1). The average right atrial (RA) area was 18.90 ± 7.24 cm^2^, and the mean RV area was 20.53 ± 6.55 cm^2^ (Table 1).

**Fig. 1:**
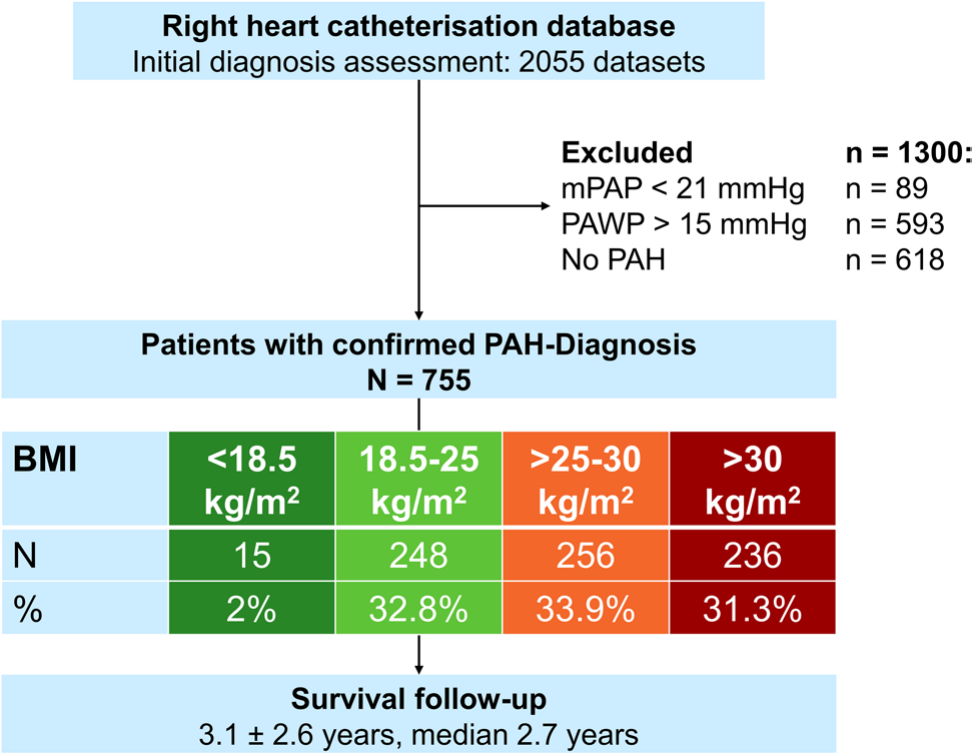
Study flow-chart. The graph provides information on patient-flow, dropouts, and BMI group assignment

In this patient cohort, there was a significant but weak correlation of BMI and CO (p < 0.0001, R = 0.232). There was no correlation of CI and BMI (p = 0.122, R = − 0.056) (Fig. 1).

### Survival

**Fig. 2:**
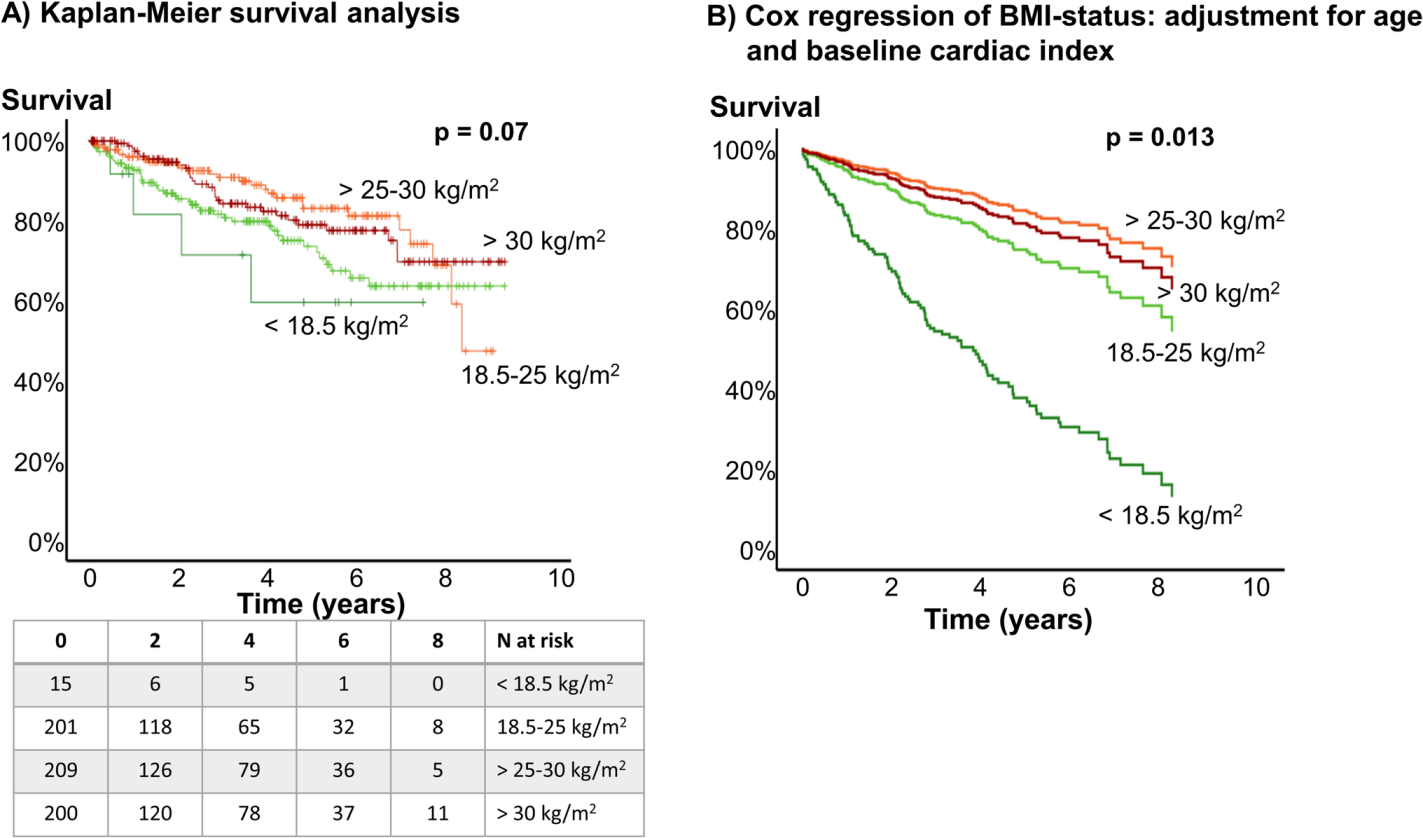
Survival analysis of different BMI groups. BMI-status significantly predicted survival in this patient cohort. Overweight and obese patients showed better survival than patients with BMI < 25 kg/m^2^ (left). When corrected for age and CI, underweight patients showed even substantially worse survival (right)

BMI groups showed a trend to predict survival in our study cohort (p = 0.07, Fig. 2). Worst survival was denoted for patients with BMI < 18.5 kg/m^2^, best survival for BMI > 25 to 30 kg/m^2^. When corrected for age and CI (Fig. 2), the difference of survival curves for patients with BMI < 18.5 kg/m^2^ became more distinct, as patients with BMI < 18.5 kg/m^2^ were predominantly younger. When comparing survival in patients with BMI < 25 kg/m^2^ and ≥ 25 kg/m^2^, patients with higher BMI had significantly better survival rates with 1-, 3- and 5-year survival rates of 96.9%, 87.5% and 81.0% vs. 91.9%, 80.1% and 69.9% (p = 0.013, Fig. 2). The difference remained significant when adjusted for age (p = 0.015), and showed a trend when adjusted for age and systolic blood pressure (p = 0.056).

In the multivariable analysis RV function (normal or mild impairment, moderate impairment and severe impairment: p < 0.0001), BMI status (p = 0.008) and sex (p = 0.011) were identified as independent prognostic predictors of age-adjusted survival. CI and CO were not identified as independent predictor of survival.

### Characteristics of different BMI groups (Table 2, Suppl. Table 1)

**Fig. 3:**
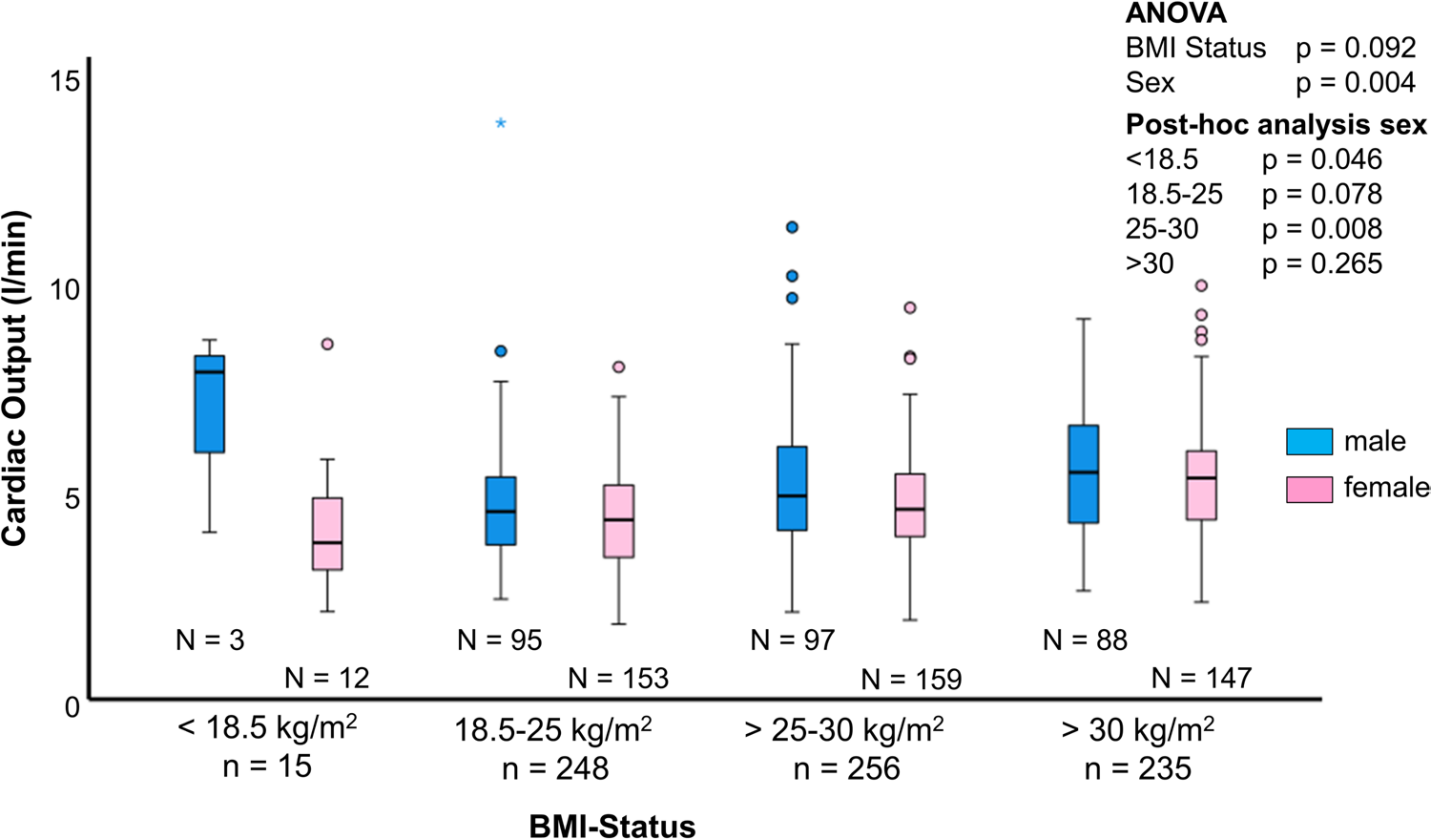
Cardiac output according to body mass index. There was a significant difference in CO between BMI groups. Patients with higher BMI showed higher CO

**Fig. 4:**
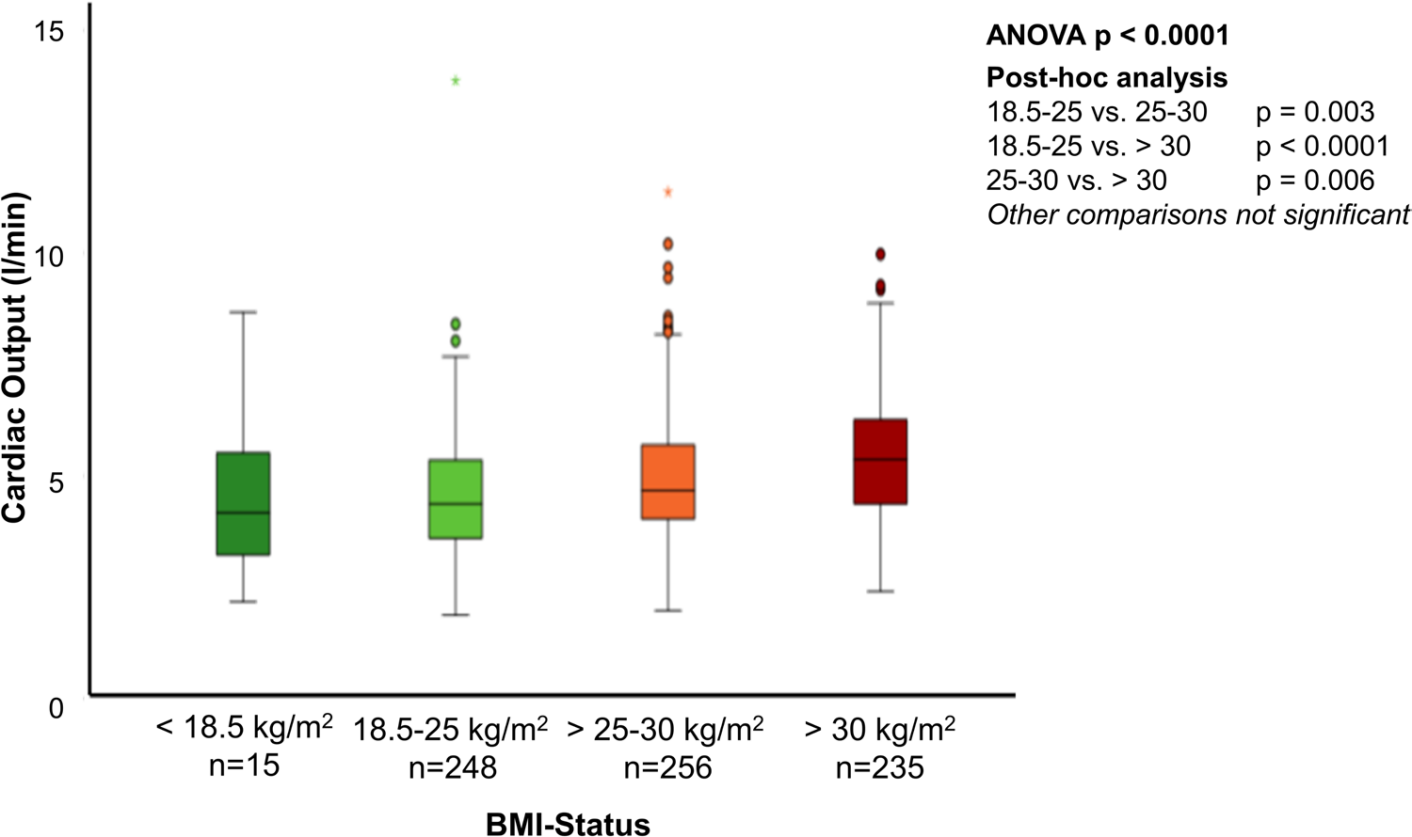
Influence of gender and body mass index on cardiac output. Sex differences were consistent within BMI groups with males having higher CO than women

BMI groups significantly differed in CO (ANOVA p < 0.0001; post-hoc tests: 18.5–25 kg/m^2^ vs. 25–30 kg/m^2^: p = 0.003; 18.5–25 kg/m^2^ vs. > 30 kg/m^2^: p < 0.0001; 25–30 kg/m^2^ vs. > 30 kg/m^2^: p = 0.006; Fig. 3, Table 2, Suppl. Table 1). Nonparametric sensitivity analysis confirmed the results (Kruskal–Wallis test p < 0.0001; post-hoc tests: 18.5–25 kg/m^2^ vs. 25–30 kg/m^2^: p < 0.001; 18.5–25 kg/m^2^ vs. > 30 kg/m^2^: p < 0.001; 25–30 kg/m^2^ vs. > 30 kg/m^2^: p < 0.001; < 18.5 kg/m^2^ vs. > 30 kg/m^2^: p = 0.020). CO significantly differed between men and women within underweight (p = 0.046) and obese patients (p = 0.008; Fig. 4). Men showed a higher CO than women (p = 0.004), with less distinct differences in groups with higher BMI. Patients with low BMI were predominantly female (< 18.5 kg/m^2^ 80% female, p < 0.0001) and younger (BMI < 18.5 kg/m^2^: 55 ± 21 years vs. 65 ± 16 years, 65 ± 14 years and 64 ± 13 years in the other BMI groups; Table 2).

Underweight patients presented with more severe symptoms (30.8% WHO FC IV vs. 10.4%, 6.9% and 9.2% in the other BMI groups, p = 0.001), significantly lower forced expiratory volume per second and forced vital capacity compared to other BMI groups (Table 2).

Obese patients had significantly larger RV area compared to normal weight (p = 0.004) and underweight (p = 0.035) patients, a higher DLCO compared to normal weight patients (p = 0.018), but significantly lower 6MWD compared to normal and overweight patients. In obese patients PAWP was significantly higher than in normal (p < 0.001) and overweight (p = 0.021) patients. The PAC of the obese group was significantly higher than underweight (p = 0.028), normal weight (p < 0.001) and overweight (p = 0.001) BMI-groups (Table 2). Obese patients also showed a significantly higher SV than underweight (p = 0.011), normal weight (p < 0.001) and overweight (p = 0.005) patients. Overweight and obese patients also showed higher TAPSE than normal weight patients (both p = 0.015).

Normal weight patients had a significantly higher NT-proBNP compared to overweight patients (p = 0.026) and presented with significantly higher PVR than the obese group (p = 0.008).

No significant distinction between the BMI-groups could be made regarding mPAP measured during RHC, sPAP measured by echocardiography, LV-EI and TAPSE/sPAP-ratio.

### Hyperdynamic cardiac output

**Fig. 5:**
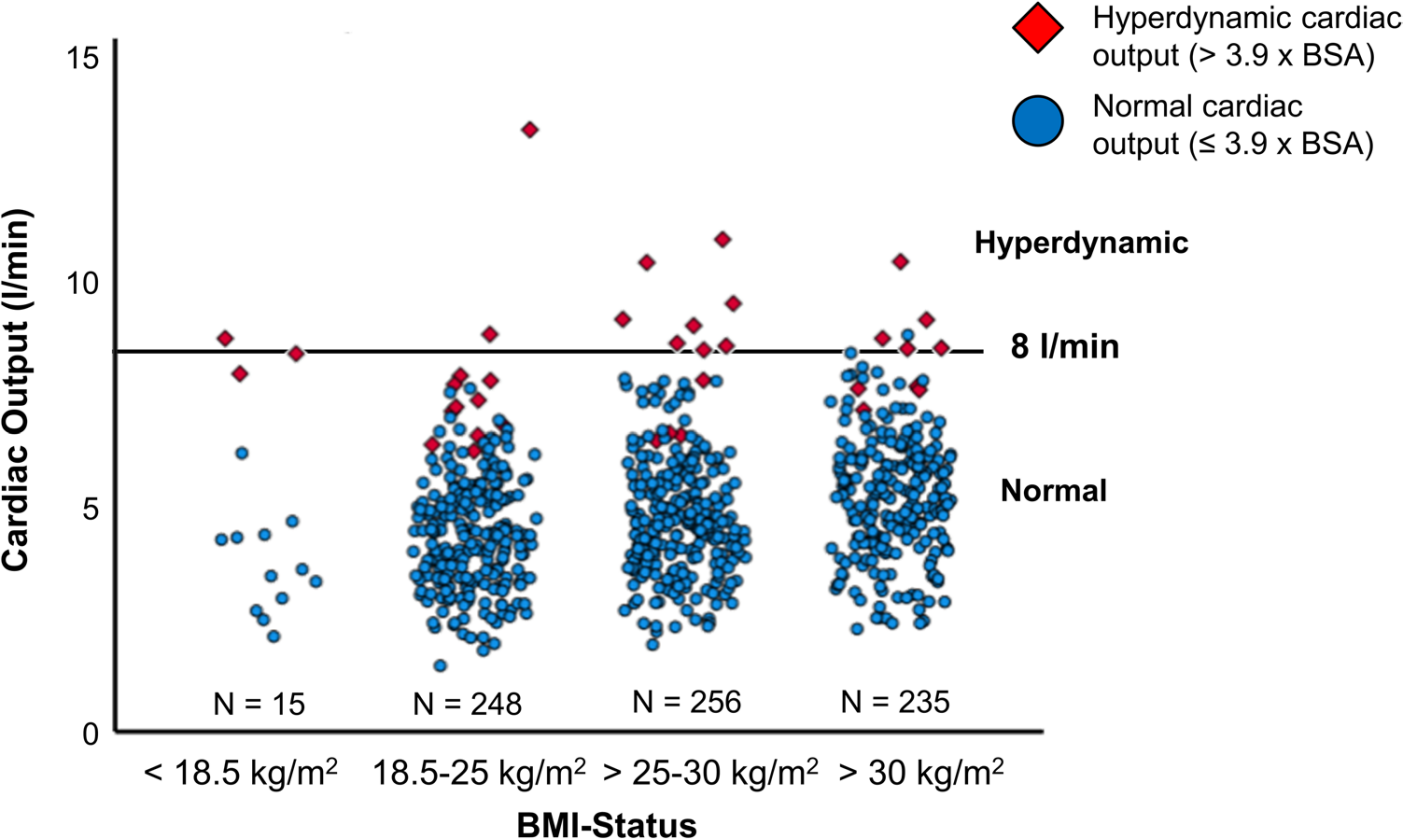
Hyperdynamic cardiac output. Hyperdynamic status was more frequent in patients with low BMI (p = 0.044) and equally distributed among other BMI groups (BMI < 18.5 kg/m^2^: 20%; vs. 4.8% in BMI 18.5–25 kg/m^2^, 4.7% in BMI 25–30 kg/m^2^ and 3.8% in BMI > 30 kg/m^2^). Different definitions of hyperdynamic status with 3.9 × BSA (indicated by diamond symbol) and > 8 l/min (circles above the horizontal line of 8 l/min) led to discordant classification in some patients

We also analysed, if obesity may lead to a hyperdynamic right ventricular pump function. Therefore, we used two different definitions of hyperdynamic status (3.9 × BSA and 8 l/min) lead to discordant classification of hyperdynamic status in some cases (Fig. 5). There was no correlation of BMI status (all further analyses with 3.9 × BSA) and frequency of hyperdynamic CO (< 18.5 kg/m^2^: 20%, 18.5–25 kg/m^2^: 4.8%, > 25 to 30 kg/m^2^: 4.7%, > 30 kg/m^2^: 3.8%), but a significant difference between groups with the highest frequency of hyperdynamic CO in patients with BMI < 18.5 kg/m^2^ (p = 0.044) (Fig. 5). Hyperdynamic patients were significantly younger (p < 0.0001), had a higher TAPSE (p < 0.0001) and a higher 6MWD (p = 0.029) than patients with CO < 3.9 × BSA. Furthermore, they showed a significantly higher glomerular filtration rate (p < 0.0001), had a higher CRP (p = 0.030), lower Troponin-T (p = 0.021) and lower urea (p = 0.018; data not shown). Hyperdynamic patients also presented with less severe PAH according to hemodynamics with lower PVR (p < 0.0001), higher PAC (p < 0.0001) and SV (p < 0.0001, data not shown).

When the definition of > 8 l/min for hyperdynamic status was used, hyperdynamic patients were taller and heavier and did not show differences in CRP, Troponin-T and urea. The other differences in clinical parameters could also be seen in patients meeting the definition of CO > 8 l/min (data not shown).

Underweight patients presented significantly more often with hyperdynamic CO (BMI < 18.5 kg/m^2^: 20%; vs. 4.8% in BMI 18.5–25 kg/m^2^, 4.7% in BMI 25–30 kg/m^2^ and 3.8% in BMI > 30 kg/m^2^, respectively). Higher frequency of hyperdynamic status in underweight patients could also be seen when only comparing patients with moderate impairment of RV function. When using the definition of hyperdynamic status with CO > 8 l/min, patients with normal weight showed significantly less often a hyperdynamic status (0.8%, vs. 13.3% in BMI < 18.5 kg/m^2^, 4.3% in BMI 25–30 kg/m^2^ and 3.8% in BMI > 30 kg/m^2^).

## Discussion

To our knowledge, this is the first study to assess RV function and hyperdynamic status in PAH by RHC and echocardiography with regard to BMI status and survival. Our survival analysis showed that PAH-patients with higher BMI of 25–30 kg/m^2^ had significantly better 1-, 3- and 5-year survival rates, even after adjustment for age. Patients with low BMI < 18.5 kg/m had the worst survival. The study underlines the importance of the parameter body weight in the clinical management of PAH patients. It provides important insights in the relations of BMI and CO and document significant gender differences with respect to right ventricular function.

### BMI and survival

The significantly better survival in PAH-patients with higher BMI of 25–30 kg/m^2^ can be described with the term “obesity paradox”, which has been observed in other forms of PH [8, 18, 19] as well as PAH-patients [20, 21] before. While the concept and existence of the obesity paradox in PH is still debated and not yet proven [10, 18, 22], this study provides an additional piece of evidence for it. However, best survival was shown for overweight, but not for obese patients, while underweight patients demonstrated most impaired survival. Apart from BMI, WHO functional class, RV function and age, but not CI were independent determining factors of survival in our study population as well.

We also noticed that obese had significantly lower NT-proBNP values and better TAPSE than patients with normal weight. Both parameters correlate with the prognosis in chronic heart failure [23, 24]. These findings suggest better right heart function among the obese individuals of our study cohort. Sharma et al. proposed that patients with high BMI have high energy reserves which enable the patient to compensate heart failure [25]. Nonetheless, there is still discordant data on obesity as a protective factor in chronic diseases and the exact mechanisms of action remain unclear.

One may speculate that overweight PAH patients may display a better body composition characterised by an optimal lean-body mass (muscle) vs. fat-body mass ratio. In alignment with this paradigm, our data reveals that patients with lower BMI values experienced a more adverse prognosis. This finding may reflect a possible status of sarcopenia, whose role in PAH is largely unknown. Such decrease in lean body mass and skeletal muscle atrophy is strongly associated with poor outcomes in left-sided heart failure [26]. Sarcopenia in PAH may also be caused by lower anabolic drive, whose effects on skeletal muscle are well-established [27].

Right ventricular function was an independent prognostic predictor of age-adjusted survival as well. This highlights the importance of the right heart in PAH. Right heart failure is a major reason for mortality in PAH and often results from an increase in right ventricular afterload. In summary high RV afterload leads to RV hypertrophy which worsens the RV perfusion. This ischemia reduces the function of the right heart which results in right heart failure eventually [28]. While it is generally observed that obese patients exhibit higher blood volume and CO, leading to increased right ventricular afterload[16], this study found that individuals with a BMI above 30 kg/m^2^ exhibited a notably higher PAC and lower PVR compared to the other BMI groups. This suggests a compensatory mechanism for the heightened blood volume, which, in turn, aids in mitigating RV afterload. Supporting this explanation are the significantly better TAPSE and NT-proBNP values measured in the obese group. These parameters are indicative of enhanced right heart function and could represent a positive adaption of the right heart, potentially contributing to an improved overall survival rate.

### Correlation of BMI and CO

The study data indicates a significant but weak positive correlation of BMI and CO in PAH patients for both males and females. This correlation between BMI and CO has already been observed in healthy subjects [4, 15–17]. A potential explanation for the observed effect could be the elevated blood volume in obese patients, stemming from not only their increased body fat but also their greater fat-free body mass. In addition, obesity also exerts its influence on the anatomy and function of the heart through various pathways resulting in hypertrophy[16]. In obese patients, there is also an enlargement of the right heart size and an increase in SV [4]. We were able to observe a significantly increased SV and RV area in obese patients as well, while heart rate remained similar across all BMI groups. It appears that the increased CO in obese patients is a consequence of the heightened SV.

### BMI and hyperdynamic right ventricular function

There was no correlation of BMI status (with 3.9 × BSA) and frequency of hyperdynamic CO. Within our study group, hyperdynamic patients exhibited superior exercise performance and more favourable indicators of right heart function (such as TAPSE and CO), as well as improved hemodynamics. This suggests that hyperdynamic patients are more effective in compensating for the burden of the disease. While Frank et al. demonstrated a positive correlation between increased BMI and mPAP [8] in PH, we were unable to replicate these results. In our cohort, we did not observe a significant difference in mPAP across the BMI-groups.

### Clinical implications and future research

Patients with low BMI < 18.5 kg/m should be closely monitored and intensively treated, as it may indicate clinical deterioration and is associated with a worse outcome. Patients with higher BMI of 25–30 kg/m^2^ may not be forced to reach normal weight as slight overweight may have some protective effect of in PAH. However, further research focusing on the existence and underlying reasons of the obesity paradox should be performed while considering other means of diagnosing obesity, such as waist-to-hip ratio or waist circumference.

## Strengths and study limitations

The study is limited by its retrospective design and respective missing values for certain clinical parameters. The recruitment could be biased geographically, because only one PH centre was used for the enrolment of patients. However, in this study a substantial cohort of real-life patients undergoing PH assessment was recruited. In addition, our cohort matches the patient characteristics of the COMPERA register [14]. Registry data indicates that approximately 30–40% of patients with PH are obese [6, 18, 29]. Our study cohort aligns with these findings, with roughly one-third of the analysed study participants falling into the BMI groups above 18.5 kg/m^2^. The underweight group, however, included only 15 patients. Consequently, interpretation of findings regarding the underweight BMI group is limited.

An additional constraint arises from the categorization based on BMI, as it lacks the ability to discern between fat-body mass and lean-body mass. Consequently, BMI fails to offer insights into the body composition, leading to potential inaccuracies in diagnosing obesity in certain cases [30]. Moreover, this study did not include data that distinguishes between patients exhibiting sarcopenic obesity and those characterised by obesity but with normal skeletal muscle atrophy. As a result, some patients might be misclassified. This is of utmost importance considering the pivotal role of muscle atrophy and function in PAH [31] and the established effects of exercise training in PAH [32–34].

There was no assessment of BMI over time, so no change in body habitus over time was recorded. However, patients suffering from acute cardiac decompensation were excluded which eliminates one possible reason for a temporary increase in body mass because of oedema [35, 36].

In addition, not all known parameters of RV function were assessed. Despite that, the parameters analysed in this study reflect the routine diagnostic work up of PH-patients.

Echocardiography as a diagnostic tool is prone to inter-observer variability [37, 38]. In our study, patient’s heart function was usually assessed via echocardiography only once by a single, yet experienced, physician. This reduces the reliability of the measurements but mimics the real-life clinical conditions. As survival analyses were of exploratory nature, respective findings have to be interpreted with caution.

## Conclusions

This is the first study to assess right ventricular function and hyperdynamic status in PAH by RHC and echocardiography regarding BMI status and survival. This study shows that patients with low BMI < 18.5 kg/m had the worst survival and should be closely monitored and intensively treated, whereas patients with slight overweight and a BMI of 25–30 kg/m^2^ had the best survival. Further studies are needed to investigate the mechanisms of action and clinical implications of the obesity paradox in PAH.

## Supplementary Information

Below is the link to the electronic supplementary material.

Supplementary file1 (DOCX 16 kb)

## Data Availability

Data is available upon reasonable request to the corresponding author.

## Abbreviations

6MWD: 6-Min walking distance
APAH: Associated pulmonary arterial hypertension
BMI: Body Mass Index
BSA: Body surface area
CI: Cardiac index
CO: Cardiac output
DLCO: Diffusion capacity of carbon monoxide
DPAH: Drugs, toxins and radiation-induced pulmonary arterial hypertension
FC: Functional class
FEV1: Forced expiratory volume per second
FVC: Forced vital capacity
HPAH: Heritable pulmonary arterial hypertension
IPAH: Idiopathic pulmonary arterial hypertension
LV-EI: Left ventricular eccentricity index
mPAP: Mean pulmonary arterial pressure
NT-proBNP: N-terminal pro brain natriuretic peptide
PAC: Pulmonary arterial compliance
PAH: Pulmonary arterial hypertension
PAWP: Pulmonary arterial wedge pressure
PH: Pulmonary hypertension
PVR: Pulmonary vascular resistance
RA: Right atrial
RHC: Right heart catheterisation
RV: Right ventricular
SD: Standard deviation
sPAP: Systolic pulmonary atrial pressure
SV: Stroke volume
SVI: Stroke volume index
TAPSE: Tricuspid annular plane systolic excursion
TDI: Tissue Doppler imaging
WHO: World Health Organization
WU: Wood Unit(s)
