# Supplementary material for: Right ventricular function in pulmonary hypertension and obesity: a cross-sectional cohort study with survival follow-up
Source: Clin Res Cardiol. 2025 Jun 24;115(3):459–71. doi: 10.1007/s00392-025-02682-9 (PMC12894162; doi:10.1007/s00392-025-02682-9)
Supplement: Supplementary file 1 — Supplementary file1 (DOCX 16 kb) [file 392_2025_2682_MOESM1_ESM.docx]

**Supplementary Table 1 Characteristics of the BMI groups**

| **BMI** | | | **<18.5** | | | | | | | | | | | | | **18.5-25** | | | | | | | | | | | | | | | **25-30** | | | | | | | | | | | | | | **>30** | | | | | | | | | | | | |
| --- | --- | --- | --- | --- | --- | --- | --- | --- | --- | --- | --- | --- | --- | --- | --- | --- | --- | --- | --- | --- | --- | --- | --- | --- | --- | --- | --- | --- | --- | --- | --- | --- | --- | --- | --- | --- | --- | --- | --- | --- | --- | --- | --- | --- | --- | --- | --- | --- | --- | --- | --- | --- | --- | --- | --- | --- | --- |
|  | | | **(N=15)** | | | | | | | | | | | | | **(N=248)** | | | | | | | | | | | | | | | **(N=256)** | | | | | | | | | | | | | | **(N=236)** | | | | | | | | | | | | |
| **Parameter [unit]** | | | N* | | Mean ± SD or n (%) | | | | | | 95%  confidence interval | | | | N* | | | Mean ± SD or n (%) | | | | | | 95%  confidence interval | | | | | | N* | | | Mean ± SD or n (%) | | | | | | 95%  confidence interval | | | | | N* | | | Mean ± SD or n (%) | | | | | | 95%  confidence interval | | | | |
| Female sex no. [%] | | |  | | 12 | |  | | (80%) | |  |  |  | |  | | | 153 | |  | | (63.9%) | |  | |  | |  | |  | | | 156 | |  | | (61.7%) | |  |  | |  | |  | | | 145 | |  | | (62.2%) | |  | |  | |  |
| Age [years] | | | 15 | | 55 | | ± | | 21 | | (44 | - | 67) | | 248 | | | 65 | | ± | | 16 | | (63 | | - | | 67) | | 256 | | | 65 | | ± | | 14 | | (63 | - | | 67) | | 236 | | | 64 | | ± | | 13 | | (62 | | - | | 66) |
| Height [cm] | | | 15 | | 164.80 | | ± | | 6.11 | | (161.42 | - | 168.18) | | 248 | | | 167.07 | | ± | | 8.66 | | (165.99 | | - | | 168.15) | | 256 | | | 167.53 | | ± | | 9.65 | | (166.34 | - | | 168.72) | | 236 | | | 167.20 | | ± | | 9.52 | | (165.98 | | - | | 168.42) |
| Weight [kg] | | | 15 | | 45.90 | | ± | | 6.30 | | (42.4 | - | 49.4) | | 248 | | | 62.60 | | ± | | 8.20 | | (61.6 | | - | | 63.70) | | 256 | | | 77.00 | | ± | | 10.00 | | (75.8 | - | | 78.20) | | 236 | | | 99.50 | | ± | | 16.50 | | (97.40 | | - | | 101.6) |
| BMI [kg/m²] | | | 15 | | 16.88 | | ± | | 1.93 | | (15.81 | - | 17.95) | | 248 | | | 22.37 | | ± | | 1.69 | | (22.16 | | - | | 22.59) | | 256 | | | 27.34 | | ± | | 1.44 | | (27.16 | - | | 27.51) | | 236 | | | 35.58 | | ± | | 5.11 | | (34.92 | | - | | 36.23) |
| BSA [m²] | | | 15 | | 1.48 | | ± | | 0.12 | | (1.41 | - | 1.54) | | 248 | | | 1.70 | | ± | | 0.15 | | (1.68 | | - | | 1.72) | | 256 | | | 1.86 | | ± | | 0.18 | | (1.84 | - | | 1.89) | | 236 | | | 2.07 | | ± | | 0.20 | | (2.05 | | - | | 2.10) |
| HR [bpm] | | | 15 | | 86.20 | | ± | | 20.26 | | (74.98 | - | 97.42) | | 248 | | | 75.08 | | ± | | 15.57 | | (73.13 | | - | | 77.03) | | 255 | | | 75.09 | | ± | | 75.09 | | (73.14 | - | | 77.03) | | 236 | | | 73.56 | | ± | | 12.89 | | (71.91 | | - | | 75.21) |
| Systolic blood pressure [mmHg] | | | 14 | | 127.29 | | ± | | 18.93 | | (116.36 | - | 138.21) | | 248 | | | 131.90 | | ± | | 21.96 | | (129.15 | | - | | 134.65) | | 255 | | | 138.69 | | ± | | 22.62 | | (135.90 | - | | 141.48) | | 233 | | | 139.32 | | ± | | 21.14 | | (136.59 | | - | | 142.05) |
| Diastolic blood pressure [mmHg] | | | 14 | | 70.21 | | ± | | 11.89 | | (63.35 | - | 77.08) | | 248 | | | 70.43 | | ± | | 9.09 | | (69.29 | | - | | 71.56) | | 255 | | | 75.29 | | ± | | 11.22 | | (73.91 | - | | 76.68) | | 233 | | | 74.53 | | ± | | 10.21 | | (73.21 | | - | | 75.85) |
| **Diagnosis** | | | | | | | | | | | | | | | | | | | | | | | | | | | | | | | | | | | | | | | | | | | | | | | | | | | | | | | | | |
|  | IPAH |  | | 8 | |  | | (53.30%) | |  | |  |  |  | | | 171 | |  | | (69.00%) | |  | |  | |  | |  | | | 180 | |  | | (70.36%) | |  | |  |  | |  | | | 181 | |  | | (76.72%) | |  | |  | |  | |
|  | HPAH |  | | 0 | |  | |  | |  | |  |  |  | | | 8 | |  | | (3.20%) | |  | |  | |  | |  | | | 7 | |  | | (2.70%) | |  | |  |  | |  | | | 3 | |  | | (1.30%) | |  | |  | |  | |
|  | DPAH |  | | 0 | |  | |  | |  | |  |  |  | | | 0 | |  | |  | |  | |  | |  | |  | | | 0 | |  | |  | |  | |  |  | |  | | | 2 | |  | | (0.80) | |  | |  | |  | |
|  | APAH |  | | 7 | |  | | (46.67%) | |  | |  |  |  | | | 69 | |  | | (27.82%) | |  | |  | |  | |  | | | 69 | |  | | (26.95%) | |  | |  |  | |  | | | 50 | |  | | (21.19%) | |  | |  | |  | |
| **Diabetes mellitus** | | |  | | 3 | |  | | (20.00%) | |  |  |  | |  | | | 18 | |  | | (7.26%) | |  | |  | |  | |  | | | 48 | |  | | (18.75%) | |  |  | |  | |  | | | 91 | |  | | (38.56%) | |  | |  | |  |
|  | Typ 1 |  | |  | |  | |  | |  | |  |  |  | | | 2 | |  | | (0.80%) | |  | |  | |  | |  | | | 1 | |  | | (0.40%) | |  | |  |  | |  | | | 4 | |  | | (1.70%) | |  | |  | |  | |
|  | Typ 2 |  | | 2 | |  | | (13.30%) | |  | |  |  |  | | | 16 | |  | | (6.50%) | |  | |  | |  | |  | | | 47 | |  | | (18.40%) | |  | |  |  | |  | | | 87 | |  | | (36.90%) | |  | |  | |  | |
|  | Typ 3 |  | | 1 | |  | | (6.70%) | |  | |  |  |  | | | 0 | |  | |  | |  | |  | |  | |  | | | 0 | |  | |  | |  | |  |  | |  | | | 0 | |  | |  | |  | |  | |  | |
| **WHO FC no [%]** | | | | | | | | | | | | | | | | | | | | | | | | | | | | | | | | | | | | | | | | | | | | | | | | | | | | | | | | | |
|  | I |  | | 0 | |  | |  | |  | |  |  |  | | | 5 | |  | | (2.20%) | |  | |  | |  | |  | | | 0 | |  | |  | |  | |  |  | |  | | | 1 | |  | | (0.50%) | |  | |  | |  | |
|  | II |  | | 4 | |  | | (30.80%) | |  | |  |  |  | | | 84 | |  | | (36.50%) | |  | |  | |  | |  | | | 93 | |  | | (39.90%) | |  | |  |  | |  | | | 57 | |  | | (26.10%) | |  | |  | |  | |
|  | III |  | | 5 | |  | | (38.50%) | |  | |  |  |  | | | 117 | |  | | (50.90%) | |  | |  | |  | |  | | | 124 | |  | | (53.20%) | |  | |  |  | |  | | | 140 | |  | | (64.20%) | |  | |  | |  | |
|  | IV |  | | 4 | |  | | 30.80%) | |  | |  |  |  | | | 24 | |  | | (10.40%) | |  | |  | |  | |  | | | 16 | |  | | (6.90%) | |  | |  |  | |  | | | 20 | |  | | (9.20%) | |  | |  | |  | |
| **Hemodynamics at rest** | | | | | | | | | | | | | | | | | | | | | | | | | | | | | | | | | | | | | | | | | | | | | | | | | | | | | | | | | |
|  | mPAP [mmHg] | 15 | | 40.00 | | ± | | 15.38 | | (31.48 | | - | 48.52) | 248 | | | 34.70 | | ± | | 12.72 | | (33.11 | | - | | 36.29) | | 256 | | | 35.69 | | ± | | 13.51 | | (34.02 | | - | 37.35) | | 236 | | | 35.46 | | ± | | 13.56 | | (33.72 | | - | | 37.20) | |
|  | PAWP [mmHg] | 15 | | 9.93 | | ± | | 3.10 | | (8.21 | | - | 11.65) | 248 | | | 9.96 | | ± | | 3.28 | | (9.55 | | - | | 10.37) | | 256 | | | 10.54 | | ± | | 2.98 | | (10.17 | | - | 10.90) | | 236 | | | 11.26 | | ± | | 2.54 | | (10.93 | | - | | 11.58) | |
|  | CO [l/min] | 15 | | 4.72 | | ± | | 2.07 | | (3.57 | | - | 5.87) | 248 | | | 4.46 | | ± | | 1.38 | | (4.28 | | - | | 4.63) | | 256 | | | 4.91 | | ± | | 1.48 | | (4.72 | | - | 5.09) | | 235 | | | 5.33 | | ± | | 1.39 | | (5.16 | | - | | 5.51) | |
|  | CI [l/min/m²] | 15 | | 3.17 | | ± | | 1.30 | | (2.45 | | - | 3.88) | 248 | | | 2.62 | | ± | | 0.75 | | (2.52 | | - | | 2.71) | | 256 | | | 2.63 | | ± | | 0.72 | | (2.54 | | - | 2.72) | | 235 | | | 2.57 | | ± | | 0.64 | | (2.49 | | - | | 2.66) | |
|  | PVR [WU] | 15 | | 7.19 | | ± | | 3.83 | | (5.07 | | - | 9.31) | 248 | | | 6.36 | | ± | | 4.74 | | (5.77 | | - | | 6.95) | | 256 | | | 5.78 | | ± | | 4.33 | | (5.25 | | - | 6.31) | | 235 | | | 5.10 | | ± | | 3.78 | | (4.61 | | - | | 5.59) | |
|  | PAC [ml/mmHg] | 15 | | 1.88 | | ± | | 1.10 | | (1.27 | | - | 2.49) | 248 | | | 2.13 | | ± | | 1.21 | | (1.98 | | - | | 2.29) | | 256 | | | 2.33 | | ± | | 1.43 | | (2.15 | | - | 2.51) | | 236 | | | 2.80 | | ± | | 1.63 | | (2.59 | | - | | 3.01) | |
| **Echocardiography at rest** | | | | | | | | | | | | | | | | | | | | | | | | | | | | | | | | | | | | | | | | | | | | | | | | | | | | | | | | | |
|  | sPAP [mmHg] | 14 | | 61.43 | | ± | | 22.82 | | (48.25 | | - | 74.61) | 238 | | | 54.55 | | ± | | 20.86 | | (51.88 | | - | | 57.21) | | 237 | | | 54.11 | | ± | | 21.53 | | (51.36 | | - | 56.87) | | 217 | | | 51.31 | | ± | | 19.78 | | (48.67 | | - | | 53.96) | |
|  | RA area [cm²] | 14 | | 16.43 | | ± | | 7.50 | | (12.10 | | - | 20.76) | 229 | | | 18.43 | | ± | | 7.93 | | (17.40 | | - | | 19.47) | | 230 | | | 19.22 | | ± | | 7.10 | | (18.30 | | - | 20.14) | | 209 | | | 19.21 | | ± | | 6.55 | | (18.31 | | - | | 20.10) | |
|  | RV area [cm²] | 14 | | 17.07 | | ± | | 5.12 | | (14.11 | | - | 20.03) | 230 | | | 19.49 | | ± | | 6.90 | | (18.59 | | - | | 20.38) | | 233 | | | 20.79 | | ± | | 6.60 | | (19.94 | | - | 21.64) | | 212 | | | 21.60 | | ± | | 5.95 | | (20.80 | | - | | 22.41) | |
|  | TAPSE [mm] | 15 | | 19.00 | | ± | | 3.78 | | (16.91 | | - | 21.09) | 228 | | | 20.42 | | ± | | 5.89 | | (19.65 | | - | | 21.19) | | 231 | | | 21.98 | | ± | | 5.94 | | (21.21 | | - | 22.75) | | 217 | | | 21.98 | | ± | | 5.84 | | (21.19 | | - | | 22.76) | |
|  | TAPSE/sPAP [mm/mmHg] | 14 | | 3.62 | | ± | | 1.81 | | (2.58 | | - | 4.67) | 225 | | | 4.42 | | ± | | 2.40 | | (4.11 | | - | | 4.74) | | 228 | | | 5.39 | | ± | | 7.09 | | (4.36 | | - | 6.31) | | 213 | | | 5.05 | | ± | | 2.48 | | (4.71 | | - | | 5.38) | |
| **Lung function** | | | | | | | | | | | | | | | | | | | | | | | | | | | | | | | | | | | | | | | | | | | | | | | | | | | | | | | | | |
|  | FVC [%] | 12 | | 62.81 | | ± | | 14.21 | | (53.78 | | - | 71.83) | 234 | | | 82.73 | | ± | | 21.68 | | (79.94 | | - | | 85.52) | | 250 | | | 80.66 | | ± | | 22.00 | | (77.92 | | - | 83.41) | | 222 | | | 78.93 | | ± | | 22.45 | | (75.96 | | - | | 81.90) | |
|  | FEV1 [%] | 13 | | 58.18 | | ± | | 20.21 | | (45.96 | | - | 70.39) | 237 | | | 78.08 | | ± | | 20.91 | | (75.41 | | - | | 80.76) | | 252 | | | 78.30 | | ± | | 22.76 | | (75.48 | | - | 81.13) | | 228 | | | 76.65 | | ± | | 22.40 | | (73.73 | | - | | 79.58) | |
|  | TLC [%] | 13 | | 95.63 | | ± | | 23.62 | | (81.36 | | - | 109.90) | 237 | | | 97.39 | | ± | | 19.63 | | (94.87 | | - | | 99.90) | | 251 | | | 93.91 | | ± | | 20.57 | | (91.36 | | - | 96.47) | | 224 | | | 94.20 | | ± | | 17.77 | | (91.86 | | - | | 96.54) | |
|  | DLCO [%] | 9 | | 41.22 | | ± | | 14.28 | | (30.24 | | - | 52.20) | 211 | | | 50.01 | | ± | | 26.25 | | (46.45 | | - | | 53.58) | | 215 | | | 52.17 | | ± | | 23.12 | | (49.06 | | - | 55.28) | | 195 | | | 57.20 | | ± | | 23.55 | | (53.87 | | - | | 60.53) | |
| **6MWD** | | | | | | | | | | | | | | | | | | | | | | | | | | | | | | | | | | | | | | | | | | | | | | | | | | | | | | | | | |
|  | 6MWD [m] | 8 | | 375.75 | | ± | | 97.13 | | (294.55 | | - | 456.95) | 157 | | | 372.75 | | ± | | 137.87 | | (351.01 | | - | | 394.48) | | 162 | | | 366.44 | | ± | | 109.86 | | (349.40 | | - | 383.49) | | 126 | | | 321 | | ± | | 116.04 | | (300.54 | | - | | 341.46) | |
| **Laboratory** | | | | | | | | | | | | | | | | | | | | | | | | | | | | | | | | | | | | | | | | | | | | | | | | | | | | | | | | | |
|  | NT-proBNP [pg/ml] | 8 | | 3439.00 | | ± | | 2819.18 | | (1082.10 | | - | 5795.90) | 210 | | | 3034.93 | | ± | | 9247.61 | | (1776.91 | | - | | 4292.96) | | 228 | | | 2025.56 | | ± | | 6102.44 | | (1229.20 | | - | 2821.91) | | 196 | | | 1175.56 | | ± | | 2155.58 | | (871.90 | | - | | 1479.22) | |
|  | GFR CDK-EPI [ml/min/1.73m²] | 15 | | 81.35 | | ± | | 36.26 | | (61.27 | | - | 101.44) | 245 | | | 73.37 | | ± | | 25.22 | | (70.20 | | - | | 76.55) | | 256 | | | 70.90 | | ± | | 23.52 | | (68.00 | | - | 73.79) | | 235 | | | 67.36 | | ± | | 24.76 | | (64.18 | | - | | 70.54) | |
|  | urea [mg/dl] | 15 | | 47.13 | | ± | | 44.49 | | (22.50 | | - | 71.77) | 248 | | | 42.70 | | ± | | 26.48 | | (39.39 | | - | | 46.01) | | 256 | | | 44.79 | | ± | | 26.74 | | (41.50 | | - | 48.08) | | 236 | | | 48.41 | | ± | | 30.59 | | (44.49 | | - | | 52.33) | |
|  | Serum creatinine [mg/dl] | 15 | | 1.16 | | ± | | 1.14 | | (0.53 | | - | 1.79) | 248 | | | 1.01 | | ± | | 0.49 | | (0.95 | | - | | 1.07) | | 256 | | | 1.05 | | ± | | 0.51 | | (0.98 | | - | 1.11) | | 236 | | | 1.12 | | ± | | 0.52 | | (1.06 | | - | | 1.19) | |
|  | TropT [µg/l] | 8 | | 21.99 | | ± | | 15.58 | | (8.96 | | - | 35.01) | 195 | | | 20.62 | | ± | | 24.31 | | (17.18 | | - | | 24.05) | | 210 | | | 18.83 | | ± | | 20.18 | | (16.09 | | - | 21.58) | | 178 | | | 18.37 | | ± | | 13.74 | | (16.34 | | - | | 20.41) | |
|  | CRP [mg/dl] | 12 | | 24.94 | | ± | | 36.60 | | (1.69 | | - | 48.20) | 121 | | | 12.76 | | ± | | 18.54 | | (9.43 | | - | | 16.10) | | 125 | | | 15.91 | | ± | | 25.17 | | (11.45 | | - | 20.37) | | 174 | | | 12.65 | | ± | | 13.55 | | (10.62 | | - | | 14.68) | |
|  | Leukocytes [/nl] | 15 | | 8.97 | | ± | | 3.98 | | (6.77 | | - | 11.18) | 248 | | | 8.15 | | ± | | 5.20 | | (7.50 | | - | | 8.80) | | 255 | | | 7.86 | | ± | | 2.69 | | (7.52 | | - | 8.19) | | 235 | | | 8.14 | | ± | | 2.37 | | (7.84 | | - | | 8.45) | |
| *sample size is given in case of missing values.  APAH: associated PAH; BMI: Body Mass Index; BSA: Body Surface Area; CI: cardiac index; CO: cardiac output; CRP: C-reactive protein; DPAH: drug and toxin associated PAH; DLCO: diffusing capacity of the lung for carbon monoxide; FEV1: forced expiratory volume in first second; FVC: forced vital capacity; GFR CDK-EPI: estimated glomerular filtration rate by Chronic Kidney Disease Epidemiology Collaboration Formula; HPAH: heritable pulmonary arterial hypertension; HR: heart rate; IPAH: idiopathic pulmonary arterial hypertension; mPAP: mean pulmonary arterial pressure; NT-proBNP: N-terminal pro brain natriuretic peptide; PAH: pulmonary arterial hypertension; PAC: pulmonary arterial compliance; PAWP: pulmonary arterial wedge pressure; PVR: pulmonary vascular resistance; RA: right atrial; RV: right ventricular; sPAP: systolic pulmonary arterial pressure; SD: standard deviation; TAPSE: tricuspid annular plane systolic excursion; TLC: total lung capacity; TropT: Troponin-T; WHO FC: World Health Organization functional class; WU: Wood Units | | | | | | | | | | | | | | | | | | | | | | | | | | | | | | | | | | | | | | | | | | | | | | | | | | | | | | | | | |
